# Using linkage analysis of large pedigrees to guide association analyses

**DOI:** 10.1186/1753-6561-5-S9-S79

**Published:** 2011-11-29

**Authors:** Seung-Hoan Choi, Chunyu Liu, Josée Dupuis, Mark W Logue, Gyungah Jun

**Affiliations:** 1 Department of Biostatistics, Boston University School of Public Health, 801 Massachusetts Avenue, Boston, MA 02118, USA; 2 Framingham Heart Study, National Heart, Lung, and Blood Institute, 73 Mount Wayte Avenue, Suite 2, Framingham, MA 01702, USA; 3 Department of Medicine, Boston University School of Medicine, 72 East Concord Street, L310, Boston, MA 02118, USA; 4 Department of Ophthalmology, Boston University School of Medicine, 72 East Concord Street, L310, Boston, MA 02118, USA

## Abstract

To date, genome-wide association studies have yielded discoveries of common variants that partly explain familial aggregation of diseases and traits. Researchers are now turning their attention to less common variants because the price of sequencing has dropped drastically. However, because sequencing of the whole genome in large samples is costly, great care must be taken to prioritize which samples and which genomic regions are selected for sequencing. We are interested in identifying genomic regions for deep sequencing using large multiplex families collected as part of earlier linkage studies. We incorporate linkage analysis into our search for Q1-associated alleles. Overall, we found that power was low for both whole-exome and linkage-guided sequencing analysis. By restricting sequencing to regions with high LOD peaks, we found fewer associated single-nucleotide polymorphisms than by using whole-exome sequencing. However, incorporating linkage analysis enabled us to detect more than half of the associated susceptibility loci (52%) that would have been identified by whole-exome sequencing while examining only 2.5% of the exome. This result suggests that incorporating linkage results from large multiplex families might greatly increase the efficiency of sequencing to detect trait-associated alleles in complex disease.

## Background

Linkage studies have fallen out of favor in recent years as genome-wide association has become the new paradigm for gene discovery. However, genome-wide association itself is perhaps reaching its limit, because the price of sequencing has decreased and is likely to drop much further. At this point, the cost of whole-genome sequencing is still high enough that great care must be taken to select which samples or genomic regions to sequence. Much of this sequencing will not include newly collected samples but will use samples from existing studies, either of the case-control or pedigree variety. We are interested in the potential of large multiplex families (with multiple affected individuals), obtained as part of linkage studies, to guide subsequent sequencing efforts. This analysis could be done either by identifying highly informative individuals to sequence, by directing the analysis to gain greater power, or by prioritizing certain regions for deep sequencing rather than taking a genome-wide approach.

In this paper, we explore the utility of linkage analysis of large pedigrees to prioritize certain genomic areas for sequencing. This method can be viewed as an extreme case of guiding an analysis for greater power [1]. Of course, any single-nucleotide polymorphism (SNP) that is strongly associated with a disease within the high-probability region would also be observed if the entire genome had been sequenced. However, if type I error rates resulting from multiple testing are appropriately accounted for, then the significance of this locus would be reduced in the whole-genome or whole-exome sequencing experimental paradigm.

In this study, we compute the variance component logarithm of odds (LOD) scores for Q1 and Q4 for all 200 replicates provided in the Genetic Analysis Workshop 17 (GAW17) data set. The median heritability for the 200 simulation replicates is 58% for Q1 and 63% for Q4. We then examine the power of the 17 truly associated Q1 SNPs by controlling the type I error inferred from the association results with 218 unassociated SNPs for Q4 because the simulation model does not include any truly associated SNPs for Q4. This allows us to compare power and type I error rates for two sequencing strategies: (1) whole-exome sequencing followed by association tests on all SNPs detected from the whole exomes and (2) targeted sequencing of exomes under linkage peaks followed by family-based association tests using polymorphisms in these linked regions.

## Methods

For each of the 200 GAW17 simulation replicates, we used the 697 individuals from 8 families for linkage and association analyses. We did not split the large multiplex families into small families. We performed genome-wide variance components linkage analysis [2] for Q1 and Q4 using the supplied identity-by-descent (IBD) information and a robust score test implemented in the R programming language [3]. We incorporated Age, Sex, and Smoking status as covariates. Note that because fully informative IBD information was provided for all 3,205 genes, there was no need to perform multipoint analyses.

For the association analysis, we computed residuals from a linear model that included Age, Sex, and Smoking status for traits Q1 and Q4 and used the residuals in subsequent association analyses. From the simulation model, we selected 17 SNPs that were truly associated with Q1. A set of 218 SNPs, including 201 SNPs that were not associated with Q1 and the true Q1 SNPs (17 SNPs), was tested for association with Q4. Each SNP was coded as 0, 1, or 2 with respect to the number of minor alleles and was used as a covariate in the RELPAL program in S.A.G.E. (version 6.0) [4]. The program is an extended Haseman-Elston regression model that incorporates correlation among relative pairs. Association analysis of the extended Haseman-Elston regression model can be written as:(1)

where *Υ_ik_* is the trait value of individual *i* in pedigree *k*, *X_ik_* is the design vector for fixed effects for individual *i* , *B* is the coefficient vector of fixed effects, *Z_ik_* is the design vector for within-pedigree random effects, *b* is the coefficient vector for pedigree-specific covariates and polygenetic effects, and the *e_ik_* are individual-specific random effects assumed to be independently and identically distributed [5]. Significance of the effects is evaluated using a Wald statistic. Using residuals of Q1 and Q4 as the dependent variables, an additive model of each SNP, and polygenic effect as covariates, we conducted association tests for all 200 replicates.

We evaluated the power for association using Q1 and type I error using Q4. Because we found unexplained genotype correlation across chromosomes, the unassociated trait Q4 was an appropriate choice to calculate the type I error. To address the problem of multiple testing, we applied two adjustments for significance thresholds using the Sidak correction:(2)

where *N* is the number of statistical tests. First, an adjustment was based on the total number of SNPs (*N* = 24,487; Sidak threshold = 2.1 × 10^−6^) analyzed in the whole exome. The second adjustment was based on the number of SNPs under the 1.5-LOD support interval for regions with a LOD score greater than 3.3 in each of the 200 replicates. We applied the significance threshold for linkage signal at a LOD of 3.3 for a conservative genome-wide significance level [6]. Because each replicate has a different 1.5-LOD support interval, the number of SNPs under the support interval is different for each simulation replicate, ranging from 0 to 1,845 (Sidak-corrected *p*-value threshold ranging from 0.05 to 2.81 × 10^−6^). *P*-values outside the LOD support area are set to 1 and are therefore never considered significant. That is, true Q1 risk alleles that are not under a support interval with a peak LOD score greater than 3.3 are not carried forward for association analysis in the linkage-guided strategy and thus are considered false negatives.

## Results

The mean size of pedigrees was 87.12 from 8 families with 202 founders and 495 nonfounders. Relative pair types consisted of 579 sib pairs, 8 half-sib pairs, 988 grandparent pairs, 1,434 avuncular pairs, and 1,840 cousin pairs. Initially, we examined the power to detect association with individual SNPs using a whole-exome sequencing paradigm. The power to detect association with the Q1 susceptibility loci using the whole-exome sequencing data is summarized in Table 1. The power was high (>80%) for two loci: C6S2981 in *VEGFA* and C4S4935 in *VEGFC.* After correcting for the number of SNPs tested in the genome-wide approach, we found that the power to detect both of these SNPs was greater than 99%. The power to detect the truly associated loci was greater than the nominal *α* level for only three of the remaining SNPs, all of them located in the *FLT1* gene (Table 1). In general, we observed that the power was largely dependent on the magnitude of effect sizes and the minor allele frequency of SNPs (Table 1).

**Table 1:** Power for association using whole-exome or linkage region sequencing

SNP	Gene	MAF	*β*	Power from WES (%)	Linked (%)	Power from LRS (%)
C4S4935	*VEGFC*	0.0007	1.35726	99.50	70.00	70.00
C1S3181	*ELAVL4*	0.0007	0.76911	0.50	0.00	0.00
C4S1873	*KDR*	0.0007	0.58301	1.00	0.00	0.00
C13S514	*FLT1*	0.0007	0.56643	0.50	0.00	0.00
C19S4831	*HIF3A*	0.0007	0.29287	1.00	1.00	0.00
C1S6540	*ARNT*	0.0014	0.24129	2.50	0.00	0.00
C13S320	*FLT1*	0.0014	0.19605	0.50	0.00	0.00
C6S2981	*VEGFA*	0.0022	1.20645	99.50	71.00	71.00
C4S1861	*KDR*	0.0022	0.56311	0.00	0.00	0.00
C4S1890	*KDR*	0.0022	0.42407	0.50	0.00	0.00
C1S6533	*ARNT*	0.0115	0.5619	0.50	0.00	0.00
C14S1734	*HIF1A*	0.0122	0.21203	0.50	0.00	0.00
C13S431	*FLT1*	0.0172	0.74136	13.50	0.00	0.00
C4S1884	*KDR*	0.0208	0.29558	3.00	0.00	0.00
C13S522	*FLT1*	0.028	0.6183	7.00	0.00	0.00
C13S523	*FLT1*	0.0667	0.64997	33.00	0.00	0.00
C4S1878	*KDR*	0.165	0.13573	4.50	0.00	0.00

*β*: estimate from association tests using a regression model; MAF: minor allele frequency; WES: whole-exome sequencing; LRS: linkage region sequencing

Next, we evaluated power using the linkage-guided paradigm. We obtained candidate loci with significant linkage peaks (LOD > 3.3) for each replicate. The number of SNPs within 1.5-LOD support intervals from the most significant linkage peaks varied substantially by replicate. Significant linkage loci were observed at all but 12 of the Q1 replicates but only at 7 of the Q4 replicates. On average, 611.7 SNPs per replicate were under linkage peaks, which represent only about 2.5% (611.7/24,487) of the exome. The percentage of the genome included in the linkage peaks varied across replicates but was never larger than 7% of the whole exome. Therefore a great reduction in sequencing cost could be achieved by restricting sequencing to areas under linkage peaks. The linkage analysis of Q4 indicates that a much smaller percentage of the exome would be sequenced for unassociated traits, with only seven replicates requiring any sequencing at all, and that none of the regions overlapped in different replicates. The average proportion of the genome sequenced for unassociated traits if sequencing were restricted to linked regions would be 11.4/24,487, or 0.04%, which suggests a low false-positive rate.

We examined the power for the true Q1 susceptibility loci using linkage results to guide our association analyses (Table 1). The two SNPs detected with high probability under the whole-exome paradigm (C6S2981 and C4S4935) were detected in the linkage analysis 71% and 70% of the time, respectively. Because the power to detect these two SNPs under the whole-exome paradigm was more than 99%, it was not surprising that these SNPs were also subsequently detected as significantly associated SNPs by the linkage-driven approach, because they were under the significant linkage intervals. Only one other true susceptibility SNP (C19S4831) was detected by the linkage-guided approach in only two replicates. However, association with this SNP was not detected in either replicate.

Seven out of 43,600 SNPs showed significant association for Q4. However, among 218 unassociated SNPs only 10 were under the linkage region, and none of these were significant. This implies that the linkage-based sequencing produced zero false positives out of 43,600 tests.

## Discussion

We examined 17 causal SNPs for Q1 and 218 unassociated SNPs for Q4. We then examined these SNPs using two sequencing paradigms: whole-exome and linkage-guided sequencing. Association results with the whole-exome sequencing approach with appropriate corrections accounting for multiple testing revealed that overall power to detect association with small effect sizes, regardless of SNP minor allele frequency, was quite low. Only two SNPs were detected with a power greater than 80%.

For the second approach, we first performed genome-wide robust variance components linkage analyses for Q1 and Q4 using the supplied IBD sharing. Then, we identified SNPs linked to traits in each replicate, defined as being within a 1.5-LOD support interval of a LOD score greater than 3.3. Finally, we recomputed the power to detect each of the Q1 SNPs under a linkage-guided sequencing paradigm, using a less stringent multiple testing penalty that accounted only for SNPs falling under linkage peaks. Using the linkage results, we detected association with the two easily detected SNPs about 70% of the time. Comparing 90% with 70% of the power to detect only 2 of the 17 susceptibility loci might seem low, but it is important to keep in mind that power to detect the other Q1 loci is also low under a whole-exome paradigm. By using the linkage-guided approach to reduce the amount of sequencing, we found that restricting sequencing under the linkage peaks would have detected more than 52% of the loci found by whole-exome sequencing despite the fact that only 2.5% as much of the genome would have to be sequenced. This statistic seems better if we restrict our attention to two loci that could be detected with high power, where restricting sequencing under linkage peaks would have detected association approximately 70% of the time. This demonstrates that sequencing under linkage peaks can be an efficient strategy for examining large multiplex families in terms of the number of true associations obtained per base pair sequenced.

## Conclusions

Our method is only the first step in an evaluation of the utility of linkage information in association analysis. It would also be important to evaluate the difference between analyses of the full sample and analyses that sequenced only families that appeared to be linked. When we examined the significance of SNPs by family, it was clear that for most SNPs the evidence for association emerged from a single family or a small group of families. Performing pedigree-specific LOD score analysis may enable a further reduction in the number of base pairs to be sequenced without compromising the power to detect mutations associated with the traits of interest. One limitation of our study is that we did not account for population substructure in our current association analyses. Further analysis would be necessary to evaluate whether or not the substructure confounds the reported findings.

## Competing interests

The authors declare that there are no competing interests.

## Authors’ contributions

SHC carried out all association analyses and power calculation, and participated in drafting the manuscript. CL carried out the linkage analyses and provided assistance with the power calculation, and participated in drafting the manuscript. JD conceived of the study, participated in the design of the study, and participated in drafting the manuscript. MWL conceived of the study, participated in its design and statistical analyses, and drafted the manuscript. GJ conceived of the study, participated in its design and statistical analyses, and helped to draft the manuscript. All authors read and approved the final manuscript.
